# OX40/OX40 ligand and its role in precision immune oncology

**DOI:** 10.1007/s10555-024-10184-9

**Published:** 2024-03-25

**Authors:** Bicky Thapa, Shumei Kato, Daisuke Nishizaki, Hirotaka Miyashita, Suzanna Lee, Mary K. Nesline, Rebecca A. Previs, Jeffery M. Conroy, Paul DePietro, Sarabjot Pabla, Razelle Kurzrock

**Affiliations:** 1 Division of Hematology and Oncology, Medical College of Wisconsin, Milwaukee, WI USA; 2 Center for Personalized Cancer Therapy, University of California San Diego, Moores Cancer Center, La Jolla, CA USA; 3 Hematology and Oncology, Dartmouth Cancer Center, Lebanon, NH USA; 4 Labcorp Oncology, Durham, NC 27560 USA; 5 OmniSeq Inc, Buffalo, NY USA; 6 MCW Cancer Center and Genomic Sciences and Precision Medicine Center, Medical College of Wisconsin, Milwaukee, WI USA

**Keywords:** OX40, Immunotherapy, Tumor necrosis factor receptor superfamily, Precision oncology

## Abstract

Immune checkpoint inhibitors have changed the treatment landscape for various malignancies; however, their benefit is limited to a subset of patients. The immune machinery includes both mediators of suppression/immune evasion, such as PD-1, PD-L1, CTLA-4, and LAG-3, all of which can be inhibited by specific antibodies, and immune-stimulatory molecules, such as T-cell co-stimulatory receptors that belong to the tumor necrosis factor receptor superfamily (TNFRSF), including OX40 receptor (CD134; TNFRSF4), 4-1BB (CD137; TNFRSF9), and glucocorticoid-induced TNFR-related (GITR) protein (CD357; TNFRSF18). In particular, OX40 and its binding ligand OX40L (CD134L; TNFSF4; CD252) are critical for immunoregulation. When OX40 on activated T cells binds OX40L on antigen-presenting cells, T-cell activation and immune stimulation are initiated via enhanced T-cell survival, proliferation and cytotoxicity, memory T-cell formation, and abrogation of regulatory T cell (Treg) immunosuppressive functions. OX40 agonists are in clinical trials both as monotherapy and in combination with other immunotherapy agents, in particular specific checkpoint inhibitors, for cancer treatment. To date, however, only a minority of patients respond. Transcriptomic profiling reveals that OX40 and OX40L expression vary between and within tumor types, and that only ~ 17% of cancer patients have high OX40 and low OX40L, one of the expression patterns that might be theoretically amenable to OX40 agonist enhancement. Taken together, the data suggest that the OX40/OX40L machinery is a critical part of the immune stimulatory system and that understanding endogenous expression patterns of these molecules and co-existing checkpoints merits further investigation in the context of a precision immunotherapy strategy for cancer therapy.

**Supplementary Information:**

The online version contains supplementary material available at 10.1007/s10555-024-10184-9.

## Introduction

Upregulation of immune checkpoints such as programmed cell death protein-1 (PD-1) and cytotoxic T lymphocyte-associated antigen-4 (CTLA-4) results in negative regulation of T-cell activation [1]. Inhibition of immune checkpoints with anti-PD-1 and anti-CTLA-4 antibodies is associated with anti-tumor response. However, benefit is limited to a subset of the patients, highlighting the need for identification of other signaling mechanism to harness anti-tumor activity by immune cells.

T-cell co-stimulatory receptors that belong to the tumor necrosis factor receptor superfamily (TNFRSF), including OX40 receptor (CD134; TNFRSF4), 4-1BB (CD137; TNFRSF9), and glucocorticoid-induced TNFR-related (GITR) protein (CD357; TNFRSF18), are potential targets for cancer immunotherapy [2, 3]. OX40 and its binding ligand OX40L (CD134L; TNFSF4; CD252) are novel immune therapeutic targets that augment the immune response. The gene for OX40 is located on chromosome 1p36 [4, 5]. OX40 expression usually peaks around 24–72 h after antigenic stimulation of the T cell receptor (TCR) by the major histocompatibility complex (MHC) [6–8]. Binding of OX40 to OX40L induces signal transduction pathways to activate immune response and regulate T-cell activation, proliferation, differentiation, expansion, and survival. Evidence also suggests a role of OX40 in Th1 and Th2 response [9, 10].

OX40 and OX40L overexpression in CD4 + T cells also has a role in the pathogenesis of multiple autoimmune diseases [11, 12]. Graft-versus-host disease (GVHD) is a common complication of allogeneic hematopoietic stem cell transplantation and is associated with significant morbidity and mortality; a preclinical study showed OX40 and O40L interaction correlated with the induction and progression of acute GVHD [13]. Studies also suggest the potential role of the co-stimulatory OX40 signaling pathway in anti-viral immune response [14, 15].

In this article, we review the impact of the co-stimulatory molecules OX40 and OX40L, including their landscape in cancers and ongoing clinical trials.

## OX40/OX40L signaling pathways and molecular mechanisms

**Fig. 1:**
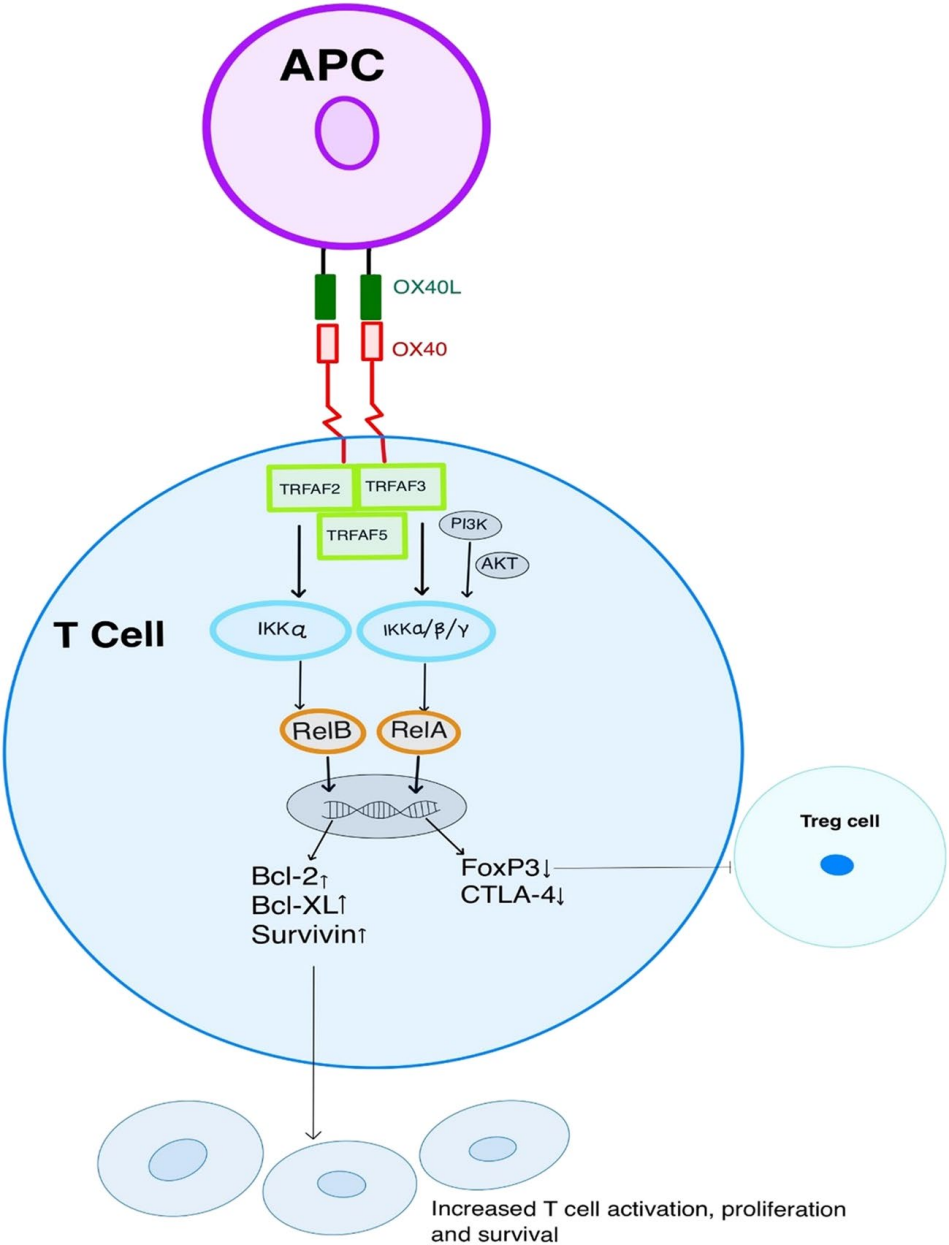
OX40/OX40L interaction with downstream signaling results in T cell activation, proliferation, and increased survival. Activated OX40 also decreases transcription of FOXP3 and CTLA-4, contributing to reduced tumor immune suppression via inhibition of Treg cells

Figure 1 illustrates the OX40-OX40L signaling pathway. The OX40 is a type 1 transmembrane glycoprotein mainly expressed by T lymphocytes. The costimulatory molecule OX40 (CD134) receptor and its ligand OX40L (CD 134L/CD252) belong to tumor necrosis factor superfamily [5]. The naïve T cell does not have OX40 and OX40L expression; however, antigenic stimulation of T-cell receptor (TCR) via the major histocompatibility complex (MHC) leads to T-lymphocyte activation, resulting in upregulation of OX40 expression [8]. Furthermore, activated T cells have upregulated CD28 expression, potentiating OX40 expression. In addition, interleukin (IL)-2 and IL-2 receptor (R) signaling is essential for satisfactory OX40 expression on activated T cells [16–18]. An *in vivo* study by Verdeil et al. demonstrated increased OX40 expression in CD8 T cells with IL-2 via STAT5-mediated signaling in the setting of weak TCR stimulation [19]. In mouse models with CD4 T cells converted CD4-CD8- double negative T cells, IL-2 facilitated the upregulated expression of OX40, supporting the survival of double negative T cells [20]. In the nonalcoholic steatohepatitis mouse model, liver tissues showed overexpression of OX40 in CD4 T cells with exogenous IL-2 stimulation [21]. The OX40 receptor is expressed on activated T lymphocytes (CD4 + and CD8 +), activated natural killer cells, regulatory T cells, neutrophils, and dendritic cells. On the other hand, OX40L is primarily expressed by antigen-presenting cells (APCs) [22]. Interaction between OX40 with OX40L is known to have immunomodulatory functions on T cell survival and proliferation. The cytoplasmic domain of OX40 is involved in downstream signaling pathways by binding with the tumor necrosis factor receptor-associated factor family (TRAF) of intracellular proteins. TRAF 2, 3, and 5 proteins are implicated in signal transduction after OX40 stimulation, mediating activation of nuclear factor-κBs (NF-κB) pathway [23, 24]. Further activation of the IκB kinase (IKK) complex and Rel A/B upregulates anti-apoptotic genes such as Bcl-2 and Bcl-XL, increasing T cells’ survival and proliferation. The activation of phosphatidylinositol-3-kinase (PI3k) and protein kinase B (PKB [Akt]) induces anti-apoptotic proteins such as Bcl-2, Bcl-xl, Bfl-1, and survivin [6, 25]. OX40 signaling also reduces expression of Forkhead box protein-3 (FOXP3) and cytotoxic T lymphocyte-associated protein (CTLA-4), contributing to decreased function of the immunosuppressive regulatory T cells [26, 27]. However, the molecular mechanism for the expression of FOXP3 and induction of Treg cells is quite complex and includes TCR signaling, cytokine milieu, transcription factors (Foxo1, STAT5, SMAD3, RUNX, NF-κB, BATF3, BATF), immune checkpoints (CTLA-4, PD-1), and costimulatory molecules [28–30]. Activation of the OX40 costimulatory receptor prevented the induction of naïve CD4 T cells to CD25 + FOX3 + T cells by inhibiting TGF-β signaling and increased cytokine production [interferon-gamma (IFN‐γ), IL-4, and IL-6] [31]. In contrast, Ruby et al. demonstrated enhanced Treg conversion with OX40 agonists by blocking cytokines, namely IFN‐γ and IL-4, in the mouse model [32]. Notably, the OX40 agonist did not affect Treg function in the *in vitro* and *in vivo* study model; it enhanced IL-2 production by CD4 + T cells, promoting increased Treg proliferation [33].

## Role of OX40 and OX40L in malignancies

OX40 is expressed on tumor-infiltrating lymphocytes (TILs) in various malignancies such as ovarian, head and neck, non-small cell lung (NSCLC), breast, colorectal, hepatocellular, and gastric cancer [34]. Studies have shown conflicting results with OX40 expression in TILs regarding clinical relevance and prognosis. Low OX40 TIL expression in tumor samples from NSCLC patients (*n* = 139) was associated with longer overall survival and better prognosis; however, the study did not specify the subtype of T lymphocytes (effector CD4 + /CD8 + T cells or CD4 + regulatory T cells) with OX40 expression [35]. The authors also found a negative correlation between PD-1 expression and TILs OX40 and OX40L expression [35]. In another study, high OX40 in tumor immune infiltrate was found to have a favorable prognosis in patients with stage I-III NSCLC (*n* = 100), but authors did not specify the subset of T cells with high OX40 expression [36]. Similarly, in patients with stage I-III colorectal cancer (*n* = 50), high OX40 expression on CD8 + T lymphocytes showed better overall survival/favorable prognosis [37]. Interestingly, advanced colorectal cancer (*n* = 22) patients with high blood levels of soluble OX40 had a worse prognosis as compared to low level of soluble OX40 [38]. In ovarian cancer, high OX40 expression on tumor-infiltrating immune cells (authors did not specify subset of T lymphocytes) correlated with longer recurrence-free survival and better response to chemotherapy [39]. Immunohistochemistry-based high expression of OX40 on breast epithelial cells and cancer cells in surgically resected specimens in patients with ductal carcinoma in situ and invasive ductal carcinoma of the breast were associated with the clinically aggressive disease; however, no differences were observed with high expression of OX40L [40]. Interestingly, high expression of OX40L in platelets from breast cancer patients was associated with high tumor grade, immune cell activation, and the tendency for metastases [41]. The tumor microenvironment (TME) in hepatocellular cancer (HCC) patients with high OX40 expression in regulatory T cells (Treg) was associated with poor survival and high serum alfa-fetoprotein level [42]. Besides, high expression of LAG3, PD-1, TIM-3, CD8, and CD68 were correlated with high OX40 expression [42]. Increased expression of OX40 on Treg cells was associated with disease progression in patients with cutaneous squamous cell carcinoma [43]. Treg cells in TME of the head and neck cancer patients demonstrated high expression of OX40, PD-1, and CTLA-4 in one of the studies but no clinical outcome was reported [44]. In patients with acute myeloid leukemia (AML), high expression of OX40 on blast cells was correlated with shorter overall survival and progression-free survival, highlighting its potential role as an immune prognostic marker, whereas no association was found with OX40L expression [45]. Additionally, high RNA expression of the *OX40 gene*/*TNFRSF4* gene from the TCGA database in AML patients was associated with *TP53*, *FLT3*, and *NPM1* mutation and unfavorable clinical outcome [46]. Taken together, the data demonstrate that expression of the OX40 machinery may correlate with either better or worse prognosis, depending on the cancer/setting studied; moreover, some studies show conflicting results. This could be potentially explained by fact that some of the studies did not specify the type of T lymphocytes with OX40 expression. Studies that specifically evaluated the Treg OX40 expression in the TME were found to be associated with poor prognosis; hence, high OX40 expression in Treg cells appears to correlate with worse clinical outcomes. Subtyping of T lymphocytes with OX40 expression is crucial as Treg plays a key role in TME in immune suppression and facilitate tumor progression [47].

## Development of drugs targeting OX40 and OX40L for cancer treatment

### Preclinical studies

Several preclinical studies demonstrated anti-tumor activity with agents targeting OX40. In four different tumor models, namely, sarcoma, breast cancer, colon cancer, and glioma, ligation of the OX40 receptor to OX40L by OX40 receptor monoclonal antibody-activated tumor T lymphocytes, resulting in enhanced anti-tumor immunity and immunological memory [48, 49]. *In vivo* genetically-engineered tumor cells via adenovirus vector facilitated gene transfer of OX40L and demonstrated anti-tumor activity in three different tumor models (lung cancer, melanoma, and colon cancer) [50]. OX40 agonist (OX40L-Fc) in a mouse sarcoma model induced anti-tumor activity due to activation and expansion of effector T cells and change in tumor microenvironment [51]. Gough et al. observed a significant increase in intra-tumoral CD8 + T cells and a decrease in Treg cells with OX40 agonist in the mouse tumor model [52]. In addition, favorable immune TME was noted with the decrease in transforming growth factor beta, myeloid-derived suppressor cells, and macrophages [52]. OX40L directed therapy with OX40L immunoglobulin conjugates in murine tumor model resulted in anti-tumor activity in a dose-dependent manner [53]. In the colon and renal cell carcinoma tumor model, treatment with Fc-mOX40L demonstrated substantial anti-tumor activity compared to OX40 agonist [54].

Piconese et al. found that Treg expressing OX40 lost their immune suppressive function by OX40 triggering and intratumor inoculation of anti-OX40 monoclonal antibody-induced complete tumor rejection via adaptive immune response [27]. In another study, OX40 stimulation by agonist (OX86 monoclonal antibody) in Treg cells attenuated the immunosuppressive function of intra-tumoral Treg cells by decreasing IL-10 production and activation of effector memory T cells [55]. The mouse tumor model by Bulliard et al. found that the OX40 agonist facilitated tumor depletion of tumor-infiltrating Treg cells by stimulating the Fcγ receptor, resulting in anti-tumor activity [56].

In the most recent study in the tumor mouse model, co-stimulation of OX40 and PD-L1 blockade in cytotoxic T cells demonstrated enhanced anti-tumor activity [57].

Promising anti-tumor activity in preclinical studies targeting OX40/OX40L paved the pathway for clinical studies in various malignancies to evaluate efficacy in real-world patients with novel agents targeting O40 costimulatory receptor, as described in the following sections.

### Early-phase clinical trials with OX40/OX40L single agent in advanced malignancies

Tables 1 and 2 outline the results from early-phase clinical trials with novel drugs targeting OX40/OX40L immune costimulatory molecules as well as clinical trials in progress.

**Table 1:** Examples of completed clinical trials targeting OX40/OX40L

Novel agent	Combination agents	Target	Phase	Cancer type	Results	NCT registry number	References
MEDI6469	Single agent, neoadjuvant	OX40	Ib	Locoregionally advanced, oral, head & neck SCC	*N* = 17, OS and DFS of 82% & 71% at 3 years	NCT02274155	[58]
MEDI6469	Cyclophosphamide, radiation	OX40	Ib	Metastatic prostate cancer	*N* = 9, clinical outcomes not available	NCT01303705	[59]
9B12	Single agent	OX40	I	Metastatic carcinoma, lymphoma, or sarcoma	*N* = 30, ORR 0%	NCT01644968	[60]
PF-04518600	Azacitadine Avelumab Glasdegib Gemtuzumab Ozogamicin	OX40	Ib/II	AML	*N* = 4, no response based on interim results	NCT03390296	[61]
SL-279252	Single agent	PD1-Fc-OX40L	I	Advanced solid tumors or lymphomas	*N* = 43, PR 1, ORR 2%	NCT03894618	[62]
PF-04518600	Utomilumab	OX40, 4-1BB	I, dose expansion cohort	Advanced solid tumors	*N* = 30 (melanoma 10, NSCLC 20); PR 1, ORR 3%	NCT02315066	[63]
Ivuxolimab (PF-04518600)	Single agent	OX40	I	Advanced or metastatic cancers	*N* = 52 PR 3 (5.8%), ORR 6%	NCT02315066	[64]
MOXR0916	Single agent	OX40	I	Advanced solid tumors	*N* = 174, 2 patients (1.1%) PR, ORR 1%	NCT02219724	[65]
MOXR0916	Anti-PD-L1	OX40	I	Advanced solid tumors	Study completed but no data available	NCT02410512	
MEDI6383	MEDI4736	OX40 ligand	I	Advanced solid tumors	*N* = 39, clinical outcomes not available	NCT02221960 (study completed but no published literature)	[66]
MEDI0562	Single agent	OX40		Advanced solid tumors	*N* = 55, ORR 4% (2/50)	NCT02318394	[67]
ATOR-1015 (Bispecific mAb)	Single agent	OX40 and CTLA-4	I	Advanced solid tumors	*N* = 15, clinical outcomes not available	NCT03782467	[68]
BMS-986178	Nivolumab (anti-PD-1), ipilimumab	OX40	I/IIa	Advanced solid tumors	*N* = 165, single agent ORR 0% (*n* = 20 patients), 0–13% ORR in combination therapy (*n* = 145)	NCT02737475	[69]
GSK3174998	Pembrolizumab	OX40	I	Advanced solid tumors	*N* = 138, No response with single agent. Combination with pembrolizumab ORR 8%; 2 CRs, 4 PRs	NCT02528357	[70]
MEDI0562	Tremelimumab (anti-CTLA-4), durvalumab (anti-PDL-1)	OX40	I	Advanced solid tumors	*N* = 58, MEDI0562 + durvalumab: 3 PR, ORR 5%	NCT02705482	[71]
INCAGN01949	Single agent	OX40	I/II	Advanced solid tumors	*N* = 87, PR 1, ORR 1%	NCT02923349	[72]
INCAGN01949	Nivolumab, ipilimumab	OX40	I/II	Advanced malignancies	*N* = 52, clinical outcomes not available	NCT03241173	
PF-04518600	With or without Axitinib	OX40	II	Metastatic renal cell carcinoma	*N* = 29, ORR 31%	NCT03092856	[73]

*AML* acute myeloid leukemia, *CR* complete response, *DFS* disease-free survival, *mAb* monoclonal antibody, *NSCLC* non-small cell lung cancer, *ORR* overall response rate, *OS* overall survival, *PR* partial response, *SCC* squamous cell carcinoma

**Table 2:** Examples of active clinical trials (currently recruiting/not recruiting) targeting OX40/OX40L

Novel agent	Combination agents	Target	Phase	Cancer type	NCT registry number
MEDI6469	SBRT	OX40	I/II	Metastatic breast cancer	NCT01862900
BMS 986178	TLR9 agonist SD-101	OX40, TLR9	I	Advanced solid malignancies	NCT03831295
BMS 986178	TLR9 agonist SD-101, radiation	OX40, TLR9	I	Low-grade B-cell lymphomas	NCT03410901
INBRX-106	Pembrolizumab	OX40	I	Locally advanced or metastatic solid tumors	NCT04198766
ES102	Toripalimab	OX40	I	Advanced solid tumors	NCT04991506
ES102	Single agent	OX40	I	Advanced solid tumors	NCT04730843
PF-04518600	Avelumab, utomilumab, ivuxolimab, radiation	OX40, 4-1BB	I/II	Advanced malignancies	NCT03217747
INCAGN01949	CMP-001	OX40, TLR9	Ib/II	Stage IV pancreatic and other cancers except melanoma	NCT04387071, study terminated as study drug no longer available
MEDI0562	Single agent	OX40	I	Head and neck squamous cell carcinoma or melanoma	NCT03336606
BGB-A445	Tislelizumab	OX40	I	Advanced solid tumors	NCT04215978
DNX-2440 (intra-tumoral injection)	Single agent	OX40 ligand	I	Resectable liver metastasis	NCT04714983
EMB-09 (bispecific antibody)	Single agent	OX40 and PD-L1	I	Metastatic solid tumors	NCT05263180
BAT6026	Anti-PD-1	OX40	I	Advanced solid tumors	NCT05109650
FS120 (bispecific antibody)	Single agent	OX40/CD137	I	Advanced malignancies	NCT04648202
HFB301001	Single agent	OX40	I	Advanced solid tumors	NCT05229601
BAT6026	Single agent	OX40	I	Advanced solid tumors	NCT05105971
HS-130	HS-110 (viagenpumatucel-L)	OX40L-Ig	I	Solid tumors	NCT04116710
PF-04518600	Avelumab, binimetinib, utomilumab, liposomal doxorubicin, or sacituzumab govitecan	OX40	II	Metastatic triple negative breast cancer	NCT03971409
IBI101	Sintilimab	OX40	I	Advanced solid tumors	NCT03758001

*SBRT* stereotactic body radiation

In the phase I clinical trial (NCT01644968), 9B12 (murine IgG1), an anti-OX40 monoclonal antibody, was evaluated for toxicity, maximum tolerated dose, and immunologic activity, respectively, and potential antitumor activity in cancer patients [60]. The study participants were divided into three cohorts (10 patients each) to receive a single cycle of an anti-OX40 monoclonal antibody with three doses on days 1, 3, and 5. Patients in cohort 1 were assigned to receive one cycle of 0.1 g/kg, cohort 2 received 0.4 mg/kg, and cohort 3 received 2 mg/kg. Patients tolerated anti-OX40 well with mostly only grade 1 or 2 toxicities. The study could not reach the maximum tolerated dose. The anti-OX40 enhanced ki-67 expression in CD4 + and CD8 + T cells at day 8 and day 15 of treatment in a dose-dependent manner, and the ki-67 level returned to the pretreatment level by day 57. The proliferating ki-67 + T cells were significantly higher in the treatment arm than in the control arm. Additional analysis showed significantly increased expression of ki-67 in CD4 + FOXP3- T cells and CD8 + T cells in patients who did not progress as compared to patients who progressed on anti-OX40 treatment. The proliferation of CD4 + FOXP3 + Treg cells did not increase with anti-OX40 treatment as compared to the control arm. Regarding efficacy, no patients achieved partial response (PR); however, regression in at least one tumor lesion was noted in 12 patients. Mixed responses were observed in two patients with renal cancer and two patients with melanoma. Interestingly, one patient with renal cancer had the most prolonged duration of response with stable disease (470 days) and received no other therapy during this period.

The phase Ib study with a murine OX40 monoclonal antibody (MEDI6469) as neoadjuvant therapy was conducted in patients with head and neck squamous cell carcinoma [58]. A total of 17 patients were enrolled in the trial, and OX40 monoclonal antibody was given at a dose of 0.4 mg/kg on days 1, 3, and 5. The treatment with neoadjuvant anti-OX40 was well tolerated, and no delay in surgery was observed. Immunologic activation after treatment with anti-OX40 was seen with an increase in CD4 + and CD8 + T cells proliferation in peripheral blood and TILs. With the median follow-up of 39 months, the overall survival in the entire cohort was 82% and disease-free survival was 71% at 3 years.

Subsequently, MEDI0562 humanized IgG, a monoclonal antibody, was developed to specifically target co-stimulatory receptor OX40 and further enhance T cell proliferation, survival, and cytokine production. The first-in-human phase I dose escalation and expansion study (NCT02318394) with MEDI0562 was conducted in patients with advanced solid tumors [67]. In total, 55 patients were enrolled and received at least one dose of MEDI562 and were evaluated for response. In the entire cohort, survival at 12 months was 47%. Two patients achieved a partial remission. The immunological study revealed an increase in peripheral CD4 + and CD8 + memory T cell proliferation and reduction in OX40 + FOXP3 + Treg cells in the tumor.

In a phase I/II clinical trial (NCT02923349), monotherapy with INCAGN01949 (fully human IgG1κ anti-OX40 agonist monoclonal antibody) was evaluated in patients with metastatic solid tumors [72]. In total, 87 patients were enrolled in the study. Treatment was well tolerated with a favorable safety profile. One patient (1.1%) with gallbladder cancer attained a partial response. After treatment with INCAGN01949, immune cell profiling did not demonstrate an increase in effector T cell proliferation or activation in peripheral blood and TILs.

SL-279252 (PD1-Fc-OX40L) is a bifunctional human fusion protein integrating the extracellular domain of PD1 and OX40 via a central Fc domain to attain simultaneous blockade of PD1 and co-stimulation of OX40. In the preclinical study, PD1-Fc-OX40L demonstrated better anti-tumor activity than PD1 blockade, OX40 agonist, or combination antibody therapy [74]. The first in human, phase 1 clinical trial (NCT03894618) with SL-279252 (PD1-Fc-OX40L) monotherapy enrolled 43 patients with advanced solid tumors or lymphomas [62]. Overall, the treatment was well tolerated. Anti-tumor activity was demonstrated in one patient with ocular melanoma, with a durable partial response. Final results from the clinical trial is pending.

Another phase I study is ongoing to evaluate bispecific antibody CTLA-4 × OX40 (ATOR-1015) in advanced solid malignancies (NCT03782467) [68].

Ivuxolimab is a fully human IgG2 monoclonal antibody OX40 agonist, which does not cause antibody-mediated cytotoxicity [64]. A phase I dose escalation study (NCT02315066) with ivuxolimab (PF-04518600) was evaluated in locally advanced or metastatic cancers [64]. Treatment was well tolerated. Three patients (5.8%) of 52 enrolled achieved partial response; one patient with melanoma who received ivuxolimab at 0.1 mg/kg (only patient in the low dose cohort with full OX40 receptor occupancy), one patient with HCC (ivuxolimab dosed at 0.3 mg/kg), and one patient with melanoma (ivuxolimab dosed at 10 mg/kg). Both melanoma patients received prior therapy with immune checkpoint inhibitors before initiation of OX40 agonist treatment. Ivuxolimab demonstrated potent immune activation, as evident by CD4 + memory T cell proliferation and activation in the peripheral blood. Additionally, tumor tissue analysis showed increased immune cell infiltration and OX40 expression. In a limited sample set, a positive association was observed between changes in tumor OX40 expression (by immunohistochemistry) and time to progression. Furthermore, RNA sequence analysis from the tumor sample at around six weeks after receiving treatment showed upregulation of genes involved in immune activation and inflammation. Interestingly, cohorts receiving lower doses of ivuxolimab (0.1 and 0.3 mg/kg) did not show immune activation; however, anti-tumor response was observed at lower doses. Potential hypothesis for low response at higher dose could be exhaustion of T cells at higher dose contributing to diminished anti-tumor response [75].

MOXR0916 is a humanized IgG1 monoclonal antibody that targets the co-stimulatory receptor OX40. In the first- in-human phase I clinical trial (NCT02219724), 172 patients with locally advanced or metastatic solid tumors received MOXR0916 [65]. About 95% of patients experienced grade 1–2 treatment-related adverse events. Two patients with renal cell carcinoma achieved a partial response. Immune activation with increased CD8 T cells and cytokines was observed in limited patients after treatment with MOXR0916.

The above-described phase I clinical trials enrolled patients with heterogeneous advanced malignancies, and most of them were heavily pretreated, including prior therapy with immune checkpoint inhibitors. The studies did not use biomarkers selection for OX40 directed treatment. More importantly, phase 1 studies mainly assessed the safety of the novel agents. In advanced malignancies, the suboptimal response with several OX40 directed therapy can also be due to complex TME with immune dysregulation and development of diverse resistance mechanism to the treatment.

Different novel agents used in the studies targeted OX40 with different pharmacokinetics and pharmacodynamics. For example, MEDI0562 was developed by humanizing a 9B12 (murine IgG1) monoclonal antibody, and INCAGN01949 is a fully human IgG1κ anti-OX40 agonist monoclonal antibody with intact Fc receptor with potential for antibody-dependent cellular toxicity to diminish intra-tumoral Treg cells. Ivuxolimab is an IgG2 agonist monoclonal antibody without antibody-dependent cytotoxicity.

### Early-phase clinical trials as combination therapy in advanced malignancies

In patients with relapsed/refractory acute myeloid leukemia, a multi-arm phase Ib/II study was conducted, and different immunotherapy combinations were evaluated. Azacitadine + venetoclax + gemtuzumab ozogamicin demonstrated appeared more active as compared to OX40 monotherapy and azacitadine + avelumab + OX40 agonist (PF-04518600) [61].

GSK3174998 is a novel humanized IgG1 monoclonal antibody agonistic specific for OX40. The phase I clinical study evaluated GSK3174998 with or without pembrolizumab in patients with advanced solid tumors (NCT02528357) [70]. In part 1 of the study, 45 patients received GSK3174998 monotherapy; no confirmed responses were observed. In part 2 of the study, 96 patients received GSK3174998 plus pembrolizumab 200 mg; an objective response rate (ORR) of 8% was observed with two complete and four partial responses.

In another phase I/IIa study, BMS-986178 (humanized IgG1 OX40 agonistic monoclonal antibody) was administered as a single agent or in combination with immune checkpoint inhibitors and evaluated for safety and efficacy in patients with advanced solid tumors (NCT02737475) [69]. No objective responses were observed in patients who received monotherapy. The ORR ranged from 0 to 13% and were noted in the cohort that received combination therapy.

In the phase I study, MEDI0562 (OX40 agonist) was given, in combination with durvalumab (anti-PDL1) or tremelimumab (anti-CTLA-4) in patients with advanced solid tumors, and demonstrated moderate toxicity after dose escalation (NCT02705482) [71]. A total of 58 patients were enrolled in the study, of which 27 received MEDI0562 + durvalumab and 31 received MEDI0562 + tremelimumab. Partial responses were observed in three patients in the MEDI0562 + durvalumab arm.

Recently, the result from a phase 2 randomized double-blind clinical trial of axitinib with or without OX40 agonist (PF-04518600) in metastatic renal cell carcinoma was presented at the 2022 ASCO GU meeting (NCT03092856) [73]. No difference in clinical outcomes was observed in patients who received axitinib plus PF-04518600 versus axitinib alone.

Preclinical studies highlighted the T cell dynamics and change in TME with OX40 agonists, resulting in anti-tumor activity in several murine tumor models. Unfortunately, the clinical trials discussed above demonstrated limited clinical response with combination treatment. A potential explanation for low activity with combination treatment could be simultaneous dosing of OX40 agonist and immune checkpoint inhibitor [75]. All trials administered OX40 agonist simultaneously with other agents such as anti-PD1/anti-PDL1. Sequential dosing of combination treatment can be considered as an OX40 agonist activating the costimulatory receptor promoting effector T cell activation, survival, and expansion. Subsequently, anti-PD1/anti-PDL1 blockade might confer better anti-tumor activity with activated effector T cells.

## Expression patterns of OX40/OX40L in cancer

Early-phase clinical trials of OX40 agonists showed anti-tumor activity in advanced solid malignancies; however, the response rate was low with single-agent as well with combination treatment. The possible reasons for low response rates include limitations of the compounds administered, accrual of patients without known OX40 expression, dysregulation of other checkpoints that could potentially attenuate efficacy, inadequate signaling of OX40 downstream pathway, and an immune suppressive microenvironment with upregulated Treg cells.

Selecting patients based on high OX40 and low OX40 ligand expression in T cells in the tumor microenvironment may warrant exploration when targeting OX40 and OX40L, as different tumors have variable levels of expression of OX40-related machinery in T cells. Various methods are used to evaluate OX40 and OX40L expression, such as immunohistochemistry and reverse transcription-polymerase chain reaction/mRNA expression, with the possibility of heterogenous results in OX40/OX40L positivity in TILs.

To evaluate the OX40/OX40L expression across diverse solid malignancies, we performed a comprehensive analysis of OX40/OX40L expression across various solid tumor types in 514 patients diagnosed with advanced malignancy at the Moore Cancer Center at the University of San Diego (Supplemental Table 1). The percentile of the OX40/OX40L expression was based on transcript level in each patient which was ranked on a scale of 0–100, and classified as low (0–24), moderate (25–74), and high (75–100) and was normalized to 735 control tumors as previously described [76–78].

**Fig. 2:**
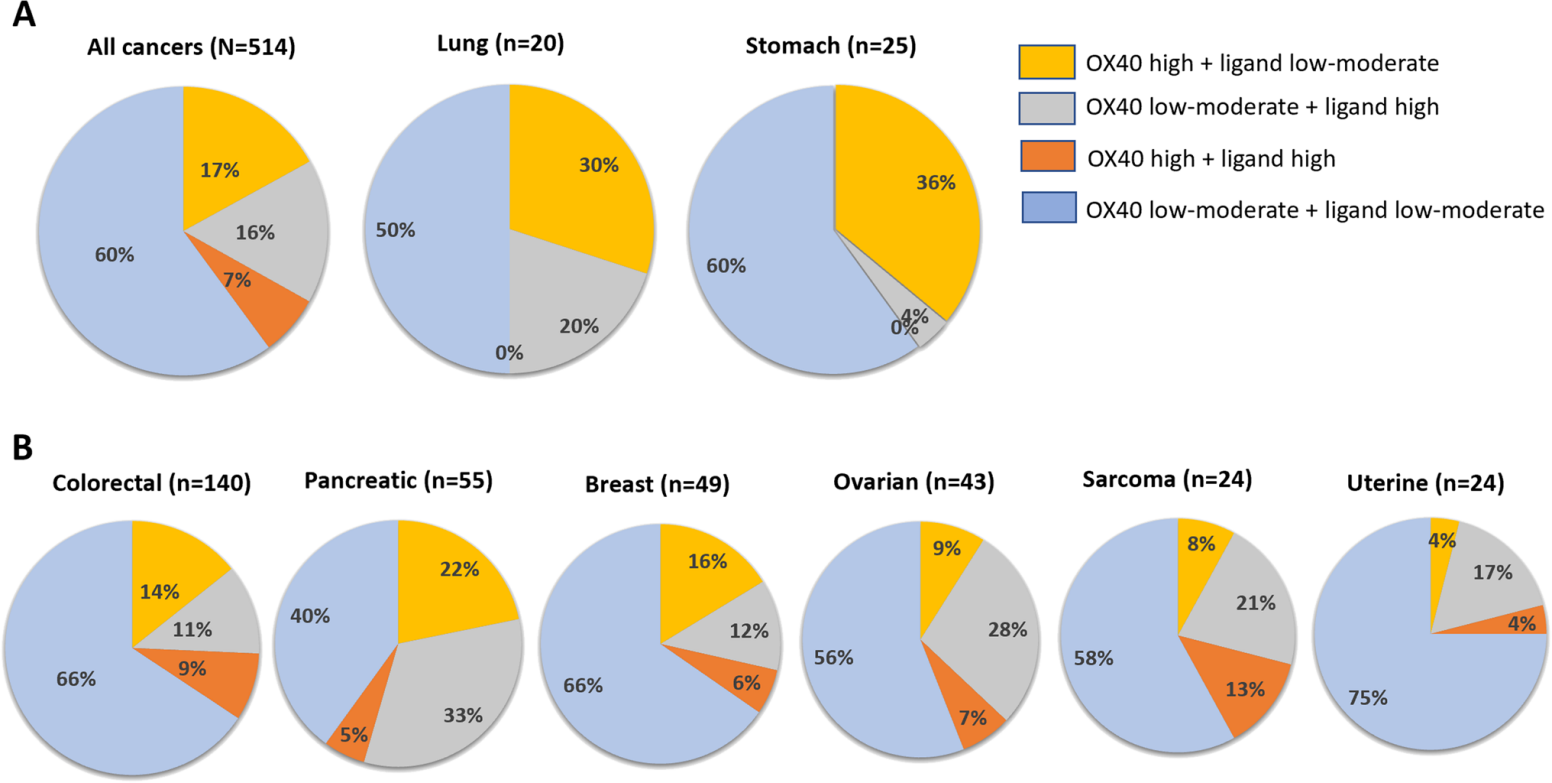
RNA expression pattern of OX40 and OX40 ligand across cancer types. **A** All cancer types and malignancies with ≥ 30% of patients with high OX40 RNA expression with low-moderate OX40L expression. **B** Different cancer types with > 20 samples. The percentile of the OX40/OX40L expression was based on transcript level in each patient which was ranked on a scale of 0–100. Classified as low (0–24), moderate (25–74), and high (75–100); low-moderate (0–74). Low defined as 0–24 percentile rank OX40/OX40L RNA expression; moderate defined as 25–74 percentile rank OX40/OX40L RNA expression; high as greater than and equal to 75–100 percentile rank OX40/OX40L RNA expression. Transcript abundance was normalized to an internal housekeeping gene profile dataset and ranked (0–100 percentile rank) in a standardized manner to a reference dataset of 735 tumors spanning across 35 tumor histologies. For descriptive purpose, expression of OX40 and OX40L was defined as follows: OX40 high + OX40 ligand low-moderate, OX40 low-moderate + OX40L high, OX40 high + OX40L high, and OX40 low-moderate + OX40L low-moderate

The percentage of high OX40 expression (≥ 75th percentile RNA rank) was 23% (118/514) across all tumor types. OX40 high expression was variable between and within cancer types; lung cancer 30% (6/20), pancreatic cancer 27% (15/55), and colorectal cancer 22% (31/140), and breast cancer 22% (11/49). For descriptive purposes (Fig. 2), we sought to assess the pattern of OX40 and OX40L expression in various tumor types such as OX40 high plus OX40L low-moderate expression, OX40 low-moderate plus OX40L high, OX40 high plus OX40L high, and OX40 low-moderate plus OX40L low-moderate. The OX40 high plus OX40L low-moderate expression pattern was observed in 17% (87/514) of all cancer types; it might be reasonable to assume that this expression pattern could be most amenable to OX40 agonist activity. This expression pattern was most common in patients with stomach (36% of patients) and lung cancer (30%) (Fig. 2).

## Conclusions and future directions

OX40, also known as CD134 or TNFRSF4, is a cell surface receptor, which is a member of the tumor necrosis factor receptor superfamily; it is found on the surface of activated T-cells, and it serves as co-stimulatory immune molecule. When T cells encounter antigens presented by pathogens or tumor cells, they become activated; OX40 is upregulated on these T cells and binds to its ligand [OX40L (CD134L; TNFSF4; CD252)], which is typically found on antigen-presenting cells, including but not limited to dendritic and B cells. The interaction between OX40 and OX40L promotes T-cell survival, proliferation, production of immunostimulatory cytokines, and maintenance of memory CD8 + T cells. Modifying the OX40-OX40L interaction can enhance the immune response to fight cancer or dampen it to treat autoimmune conditions [79]. Many experimental drugs that serve as OX40 agonists are in cancer clinical trials. OX40 agonists are being explored as monotherapy and in combination with other immunotherapy agents for cancer treatment. To date, however, while responses have been observed, they remain isolated to a minority of patients. PDL1 expression, high tumor mutational burden (≥ 10 mutations/megabase), and high microsatellite instability (MSI-H) are well known biomarkers that are associated with improved outcomes with immunotherapy [80–84]. PD1 expression on tumor-infiltrating lymphocytes has also been recently shown to correlate with better outcome after anti-PD1/PDL1 agents are given to patients with cancer [85]. Results from a recent meta-analysis of clinical trials (over 19,000 patients) found that most immune-oncology clinical studies did not include biomarkers for patient selection, even though retrospective analysis showed that biomarkers were independently correlated with improved immunotherapy outcome [86]. Therefore, the role of OX40 and OX40L expression and its association with expression of other checkpoints and biomarkers such as TMB, MSS, PD1, PDL1, and LAG3 warrants exploration in the context of determining if patient selection for monotherapy OX40 agonists and for combination therapy with specific checkpoint inhibitors would enhance response rates and other outcome parameters. The role of OX40 might also be complicated as OX40 expression on Tregs may be immunosuppressive.

Transcriptomic profiling reveals that OX40 and OX40L expression is variable between tumors and that the pattern of high OX40 and low OX40L, which might theoretically be most amenable to OX40 agonist compounds, occurs in only 17% of cancer patients, most commonly in lung and breast cancers. The correlation between OX40 protein expression and RNA expression however remains unclear and merits investigation in future studies. A precision immune oncology approach, interrogating individual tumor expression patterns of OX40 and OX40L and other immunomodulatory effectors, may warrant exploration in future studies of both single-agent and combination regimens with OX40 agonists.

### Supplementary Information

Below is the link to the electronic supplementary material.

Supplementary file1 (DOCX 12 KB)

## Data Availability

The datasets used and/or analyzed during the current study are available from the corresponding author on reasonable request.
