# Supplementary material for: OX40/OX40 ligand and its role in precision immune oncology
Source: Cancer Metastasis Rev. 2024 Mar 25;43(3):1001–13. doi: 10.1007/s10555-024-10184-9 (PMC11300540; doi:10.1007/s10555-024-10184-9)
Supplement: Supplementary file 1 — Supplementary file1 (DOCX 12 KB) [file 10555_2024_10184_MOESM1_ESM.docx]

**Supplemental Table 1: Patient Characteristics**

| **All cancers** | N = 514 |
| --- | --- |
| Median Age (range) years | 61 (24-93) years |
| Men | 40% (N = 204) |
|  |  |
| **Tumor Histology** | **Number of patients (%)** |
| Colorectal cancer | 140 (27%) |
| Breast cancer | 49 (10%) |
| Pancreatic cancer | 55 (11%) |
| All others (<10 samples per histology) | 46 (9%) |
| Ovarian cancer | 43 (8%) |
| Stomach cancer | 25 (5%) |
| Sarcoma | 24 (5%) |
| Uterine cancer | 24 (5%) |
| Lung cancer | 20 (4%) |
| Liver and Bile duct cancer | 19 (4%) |
| Esophageal cancer | 17 (3%) |
| Neuroendocrine cancer | 15 (3%) |
| Unknown Primary | 13 (2%) |
| Head and neck cancer | 12 (2%) |
| Small intestine cancer | 12 (2%) |
